# Long-term effects of aviary management and dietary fiber during pullet rearing on performance, egg quality, integument, and keel bone condition in extended laying cycles

**DOI:** 10.1016/j.psj.2026.107859

**Published:** 2026-09-29

**Authors:** R. Schreiter, S. Becker, L. Fitz, J. Krieg, P. Schulte-Huxel, A. Schröder, J. Slama, H. Louton, P. Hofmann

**Affiliations:** a Martin Luther University Halle-Wittenberg, Theodor-Lieser-Straße 11, Halle, 06120, Germany; b Bavarian State Research Center for Agriculture, Kitzingen, Germany; c Chamber of Agriculture of North Rhine-Westphalia, Bad Sassendorf, Germany; d University of Rostock, Rostock, Germany; e Ludwig Maximilian University Munich, Munich, Germany

**Keywords:** Pullet rearing, Dietary fiber, Aviary management, Integument, Extended laying period

## Abstract

Extending the laying period is a key objective in modern egg production, as it reduces resource use and improves economic efficiency. Pullet rearing can exert long-term effects on welfare and performance, but little is known about whether specific, practically applicable rearing interventions have persistent and trait-specific effects throughout extended laying cycles.

The aim of this study was to investigate long-term effects of dietary fiber during pullet rearing and aviary opening time on welfare and performance during an extended laying period of 84 weeks. In a 2 × 2 factorial design, non–beak-trimmed pullets (Lohmann Brown Classic) received either a standard (Fcon) or moderately elevated fiber diet (F+) and aviary levels were opened early (day 14/17; M14) or late (day 28/31; M28). After the controlled rearing experiment, 1,520 hens were housed under identical aviary conditions in 16 pens until 84 weeks of age, allowing persistent effects of the rearing treatments to be evaluated during an extended laying cycle. During lay, plumage damage (PD), skin injuries (SI), keel bone deviations (KBD), and keel bone fractures (KBF) were assessed at four time points. Laying performance, feed intake, mortality, and egg quality traits were also recorded.

Binary logistic regression showed lower prevalences of PD, SI, and KBD in M14 compared with M28 (P ≤ 0.004). Increased dietary fiber during rearing was associated with lower KBD but higher PD (P ≤ 0.042). Treatments had no effects on laying performance or feed intake (P > 0.05), but affected albumen consistency at 80 weeks of age, with higher values in M14 compared with M28 (P = 0.002). Survival analysis showed no effect of aviary opening time (P = 0.591), but a significant effect of diet (P = 0.015), with higher survival probability in Fcon than in F+.

Earlier aviary opening had persistent beneficial effects on integument and keel bone condition, whereas increased dietary fiber provided no consistent long-term benefit. Thus, rearing interventions can have trait-specific carry-over effects into extended laying cycles.

Scientific section Animal Well-Being and Behavior

## Introduction

Extending the productive lifespan of laying hens has become a key objective in modern egg production, as longer production cycles increase the number of eggs produced per reared pullet, reduce flock replacement, and thereby improve production efficiency and sustainability (Bain et al., 2016; Preisinger, 2021). Extended laying cycles are generally defined as production periods beyond the conventional flock replacement age of approximately 70–75 weeks of age (WA) (Bain et al., 2016; Preisinger, 2021). Recent reviews indicate that laying performance and egg quality can be maintained at acceptable levels beyond 70 WA, although between-flock variation increases with age (Arulnathan et al., 2024). In alternative housing systems, production cycles of approximately 80–85 WA for brown layers and 85–95 WA for white layers without molting are considered realistic targets (Damme et al., 2023).

Achieving such production cycles, however, requires adapted management to maintain animal health, low mortality, and egg quality, particularly eggshell strength and albumen quality (Damme et al., 2023; Arulnathan et al., 2024). In addition, age-related deterioration of welfare indicators, including plumage damage (PD), skin injuries (SI), and keel bone damage, represents a major challenge in prolonged production (Nicol et al., 2013; Rodenburg et al., 2013; Spindler et al., 2016; Li et al., 2024). Consequently, management strategies that promote long-term health and welfare are essential for the successful implementation of extended laying cycles. As extended laying cycles place greater demands on the long-term health and resilience of laying hens, management during the pullet rearing phase is expected to become increasingly important.

Pullet rearing is considered a key factor for successfully extending the productive lifespan of laying hens (Schreiter et al., 2020a, 2022; Damme et al., 2023). Conditions during this early life stage influence behavioral development, locomotor coordination, and bone growth, thereby shaping the birds’ ability to adapt to complex housing systems later in life (Janczak and Riber, 2015; De Haas et al., 2014). Several studies have shown that management measures applied during rearing may have long-term effects on welfare and performance traits during the laying period under both commercial and experimental conditions (Gilani et al., 2013; Casey-Trott et al., 2017; Schreiter et al., 2020a; Rentsch et al., 2024). These so-called carry-over effects emphasize that management and nutritional strategies applied during early life may have lasting consequences for welfare and performance traits, thereby playing a crucial role in the stability of extended laying cycles.

In northwestern Europe, laying hens are predominantly kept in multi-tier aviary systems, and pullet rearing is increasingly adapted to these systems to facilitate a smooth transition to the later housing environment (Heerkens et al., 2016; Lohmann Breeders, 2021). In aviary rearing systems, chicks usually remain on the wire tiers during the first weeks of life, while access to the littered scratching area is only possible after the aviary levels are opened. Early access to litter and environmental enrichment provides opportunities for exploratory and foraging behaviour and may reduce the development of feather pecking by allowing chicks to express species-specific behaviours during a sensitive developmental period (Janczak and Riber, 2015; Zepp et al., 2018; Tahamtani et al., 2016). Likewise, early exposure to a structurally complex environment may promote locomotor development, spatial orientation, and bone adaptation, thereby improving the ability of pullets to use multi-tier systems later in life (Casey-Trott et al., 2017). Although practical recommendations suggest opening the aviary levels at approximately three to five WA (Pottgüter et al., 2018), little is known about how the exact timing of aviary opening during rearing affects welfare and skeletal health throughout the subsequent laying period. To our knowledge, no longitudinal study has specifically investigated whether the timing of opening an otherwise comparable rearing aviary has persistent effects on integument and keel bone condition into an extended laying cycle.

Besides the housing environment, feeding also plays an important role in the occurrence of feather pecking and cannibalism. Diets with increased fiber content can prolong feeding time, stimulate foraging behavior, and promote the development of the gizzard (Hartini et al., 2002; van Krimpen et al., 2005; Svihus, 2011). Together with environmental enrichment, nutritional strategies that stimulate exploratory and foraging behaviour are considered promising approaches for preventing feather pecking during rearing (Zepp et al., 2018; Mens et al., 2020). Dietary fiber during rearing has previously been investigated in relation to feeding behavior and feather damage as well as gastrointestinal development and subsequent productive performance (van Krimpen et al., 2009; Guzmán et al., 2016). However, the long-term consequences of a moderately increased fiber supply specifically during pullet rearing for integument condition, keel bone health, and survival after transition to a common laying diet remain poorly understood, particularly during extended laying cycles.

Previous longitudinal studies therefore provide important evidence that rearing conditions can affect feather pecking, plumage condition, and keel bone health later in life. However, these studies have primarily investigated different rearing environments, early litter or enrichment provision, or different levels of environmental complexity, and follow-up generally ended no later than 70 WA (Gilani et al., 2013; Tahamtani et al., 2016; Casey-Trott et al., 2017; Schreiter et al., 2020a; Rentsch et al., 2024). Moreover, available evidence includes both observational studies of commercial flocks and controlled experiments in which hens were not maintained in aviary systems throughout both rearing and lay. The present study addresses these gaps by combining two defined and practically applicable rearing interventions in a controlled 2 × 2 factorial design, followed by identical aviary housing and management during lay up to 84 WA. This approach allows persistent and potentially trait-specific carry-over effects of rearing management and nutrition to be evaluated under conditions relevant to extended commercial laying cycles.

Therefore, the aim of the present study was to investigate the effects of dietary fiber content during pullet rearing and the timing of aviary opening on plumage damage, skin injuries, keel bone deviations (KBD), and keel bone fractures (KBF) in laying hens during the laying period, as well as potential long-term effects on performance traits, mortality, and egg quality parameters. It was hypothesized that earlier aviary opening during rearing and an increased dietary fiber content would reduce the prevalence of PD, SI, and keel bone damage during the laying period without negatively affecting performance traits.

## Materials and methods

### Experimental design and study background

The present study is part of a longitudinal experiment investigating the effects of aviary management and feeding strategies during pullet rearing on animal welfare indicators during the subsequent laying period. The study design and the results of the rearing phase have been described in detail previously by Schreiter et al. (2026).

During rearing, two factors were varied in a 2 × 2 factorial design: the fiber content of the rearing diet and the timing of aviary opening. Non–beak-trimmed pullets of the hybrid Lohmann Brown Classic (Lohmann Breeders, Cuxhaven, Germany) were reared in an aviary system at the Bavarian State Estate, Experimental and Educational Center in Poultry, located in Kitzingen, Germany. The aviary levels were opened either early (day 14 for the lower level and day 17 for the upper level; variant M14) or later (day 28 and day 31, respectively; variant M28). At the same time, birds received either a rearing diet with a fiber content typically used under practical conditions (Fcon; 4.9% crude fiber in the grower diet and 5.8% in the developer diet) or a diet with moderately increased fiber content (F+), formulated to provide approximately 20% more aNDFom than the control diet, resulting in crude fiber contents of approximately 6.8% and 7.8%, respectively. The combination of both factors resulted in four study variants (M28_Fcon, M28_F+, M14_Fcon, and M14_F+ - see Schreiter et al., 2026).

The higher fiber content of the F+ diets was achieved primarily by increasing wheat bran and lignocellulose (OptiCell®), with additional adjustments in rapeseed expeller and sunflower meal. The F+ diets were formulated to provide approximately 20% more aNDFom than Fcon. Full diet composition and nutrient analyses are provided in Schreiter et al. (2026).

After completion of the rearing phase, 1,520 pullets were transferred to a laying house equipped with an aviary system at the Chamber of Agriculture of North Rhine-Westphalia, Bad Sassendorf, Germany, where all hens were kept under identical housing and management conditions. Birds from each rearing pen were transferred together to one laying pen, thereby maintaining the original pen structure. The four experimental variants were each replicated in four identical pens (n = 4 per treatment), resulting in a total of 16 pens, with 95 hens housed per pen. The pens were divided into four sections each representing one experimental block according to their location in the hen house. The present study focuses on the laying phase and investigates whether the feeding and management strategies applied during rearing have long-term effects on integument condition and keel bone traits during an extended laying period.

### Ethical statement

The animals were housed in compliance with the applicable legal standards of the European Union (Council Directive 1999/74/EC on the minimum standards for the protection of laying hens, 1999) as well as German national regulations (German Order on the Protection of Animals and the Keeping of Production Animals, 2006). The study design was reviewed by the Animal Welfare Officer of the Bavarian State Research Center for Agriculture and was not classified as an animal experiment requiring approval.

### Animals, housing and management during the laying period

At 17 WA, the hens were transported to the laying facility in Bad Sassendorf, Germany, where they were housed under identical management and housing conditions throughout the laying period.

The laying period, with an observation period until 84 WA, was conducted in a facility equipped with a two-tier aviary system (Natura 60, Big Dutchman AG, Vechta-Calveslage, Germany), comprising 16 structurally identical compartments (each 13.6 m² in total area, consisting of 8.20 m² slatted flooring and 5.42 m² littered area). Based on the total usable space offered in the aviary compartments (slatted plus littered area), the stocking density was 7 hens/m². The floor of the littered area was covered with straw pellets (Wegmann Agrar Pellets, Rheine, Germany).

The following equipment was available to the hens within the aviary system: one nipple drinker per 10 hens (TopNippel, Big Dutchman AG, Vechta-Calveslage, Germany), 10 cm feeder space per hen provided by a flat chain feeder (Big Dutchman AG, Vechta-Calveslage, Germany), 120 cm² nest area per 95 hens (colony family nest, Big Dutchman AG, Vechta-Calveslage, Germany), and 19 cm of round metal perch per hen (Big Dutchman AG, Vechta-Calveslage, Germany).

As additional enrichment material, the hens of all groups had *ad libitum* access to pecking stones (Vilolith medium, Deutsche Vilomix Tierernährung GmbH, Neuenkirchen-Vörden, Germany) and miscanthus briquettes (Miscanthus-Brix, Miscanthus OppStock GbR, Stemwede, Germany) or spelt husk pellets (Dinkelspelzen-Briketts, Agrar-Pool GmbH, Neubrandenburg, Germany) provided in small racks. In addition, the hens were supplied once per week with grit (Geflügel-Magenkies, Raiffeisen, Hellweg-Lippe, Germany) with particle sizes of 2–5 mm and 4–7 mm.

After housing, the hens were initially fed a pre-lay diet (weeks 18–19; l-Premium Vorlege, BEST 3 Geflügelernährung GmbH (Twistringen, Germany)). Subsequently, a four-phase feeding program from the feed manufacturer BEST 3 Geflügelernährung GmbH (Twistringen, Germany) was applied. This consisted of a laying hen diet (period 1) from weeks 20–28 (L-Premium 1; 11.3 MJ ME/kg, 17.0% CP, 5.0% crude fibre, 3.5% calcium) a laying hen diet (period 2) from weeks 29–48 (L-Premium 2; 11.3 MJ ME/kg, 16.5% CP, 5.0% crude fibre, 3.5% calcium), a laying hen diet (period 3) from weeks 49–68 (L-Premium 3; 11.3 MJ ME/kg, 16.0% CP, 5.0% crude fibre, 3.7% calcium), and a laying hen diet (period 4) from weeks 69–84 (L-Premium 4; 11.2 MJ ME/kg, 15.5% CP, 5.0% crude fibre, 3,9% calcium). Nutrient concentrations represent declared values provided by the manufacturer and are expressed on an as-fed basis.

Lighting management followed a step-up lighting regime based on established management recommendations (Lohmann Breeders, 2021), with hens receiving 14 hours of light per day. A vaccination protocol appropriate to the flock’s use, geographic location, and health status was applied throughout the laying period in accordance with established guidelines (Lohmann Breeders, 2021) to maintain flock health.

### Data collection

#### Production traits

During the laying period, the number of total eggs and floor eggs (i.e., found in the litter) were recorded daily. Feed intake was recorded for each pen during the laying period. Feed conversion ratio was calculated as kilograms of feed consumed per kilogram of egg mass produced. The laying performance was calculated per average hen and per hen housed. Eggs were sorted (sorting machine, Moba Typ 6, hourly output 7900 eggs, Barneveld, Netherlands) according to their egg size and whether they were cracked, conforming to the Commission Regulation (EC, No.2023/2464; XL – individual egg mass ≥73 g, L – 63 g ≤ individual egg mass <73 g, M – 53 g ≤ individual egg mass <63 g, S – individual egg mass <53 g). Egg quality traits were assessed with 44 WA, 56 WA, 68 WA and 80 WA with randomly selected 120 eggs per treatment each. The breaking strength of the egg shells (FEST V2.0 egg shell tester, Futura, Lohne, Germany), and the albumen height (1/A 2001 albumin altimeter, Futura, Lohne, Germany) were measured. Based on the egg weight and albumen height, and according to Haugh (1937), the Haugh unit (Haugh unit = 100log [albumen height − 1.7 × egg weight0.37 + 7.69]) for characterizing albumen consistency were calculated.

Animal losses were recorded daily. Dead hens were examined for signs of cannibalism. Following the classification described by Schreiter et al. (2020b), birds showing bloody skin lesions or blood-stained plumage surrounding skin injuries were classified as cannibalism-related mortality.

#### Integument and keel bone condition

To indirectly assess the occurrence of severe feather pecking and cannibalism within the flock, integument condition was evaluated as an established animal-based indicator (Schwarzer et al., 2022). The determination of the required sample size followed the methodological approach described by Schreiter et al. (2020a), with individual hens considered as observational units for animal-based assessments. Sample size estimation was performed using a power analysis based on preliminary data (not shown; calculations conducted using https://imsiewebarchiv.uni-koeln.de/beratung/rechner/ps.html). To detect a difference of 15% in the proportion of birds exhibiting integument damage with a statistical power of 0.80 and a significance level of α = 0.05, a minimum of 113 hens per treatment group was required.

During the laying period, scoring was performed at four time points, namely at 20, 40, 60, and 80 WA. At each assessment, 30 hens per pen were randomly selected and evaluated. These birds were sampled from different functional areas of the aviary system, including the lower aviary level (n = 7), the upper aviary level (n = 7), the middle scratching area (n = 8), and the outer scratching area (n = 8). Consequently, 120 hens per treatment group were examined at each scoring date. The integument and keel bone assessments were conducted by two observers who had previously undergone intensive training involving 500 birds. In addition, inter-observer reliability was evaluated by means of a comparison of scoring results from 60 birds (see Statistical analyses for calculation).

The assessment of integument traits – PD, SI, and foot pad swelling – as performed in accordance with the Welfare Quality® (2009) protocol. Each of these traits was classified using a three-point ordinal scoring scale. PD was categorized as 0 = intact plumage, 1 = moderate PD with one or more featherless areas ≤5 cm in diameter, and 2 = severe PD with one or more featherless areas >5 cm in diameter. SI were scored as 0 = intact skin, 1 = mild skin lesions with one or more small injuries ≤1 cm, and 2 = severe skin lesions with one or more injuries >1 cm. Foot pad swelling was classified as 0 = feet intact with no or minimal epithelial proliferation, 1 = necrosis or epithelial proliferation or chronic bumblefoot without or with moderate swelling, and 2 = pronounced swelling visible dorsally.

Plumage condition was assessed separately for the back, the belly (including the cloacal region and the ventral rump), and the dorsal neck. In addition to the individual scores assigned to these three body regions, an overall plumage score was calculated for each bird by summing the respective regional scores (Schreiter et al., 2020a). For graphical presentation, the stacked bar charts (Figure 1) represent the mean value of the three plumage regions for each score category.

**Figure 1:**
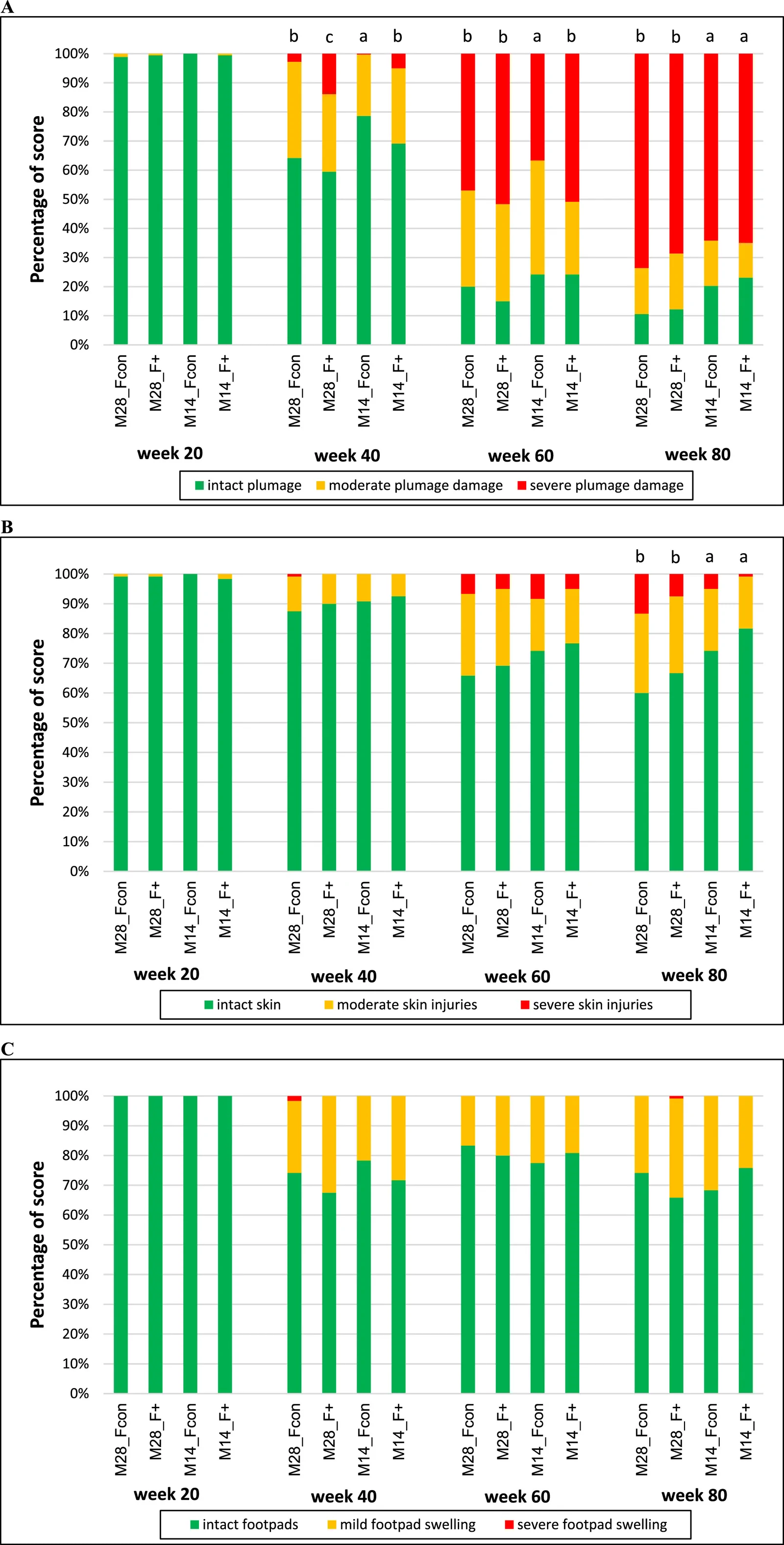

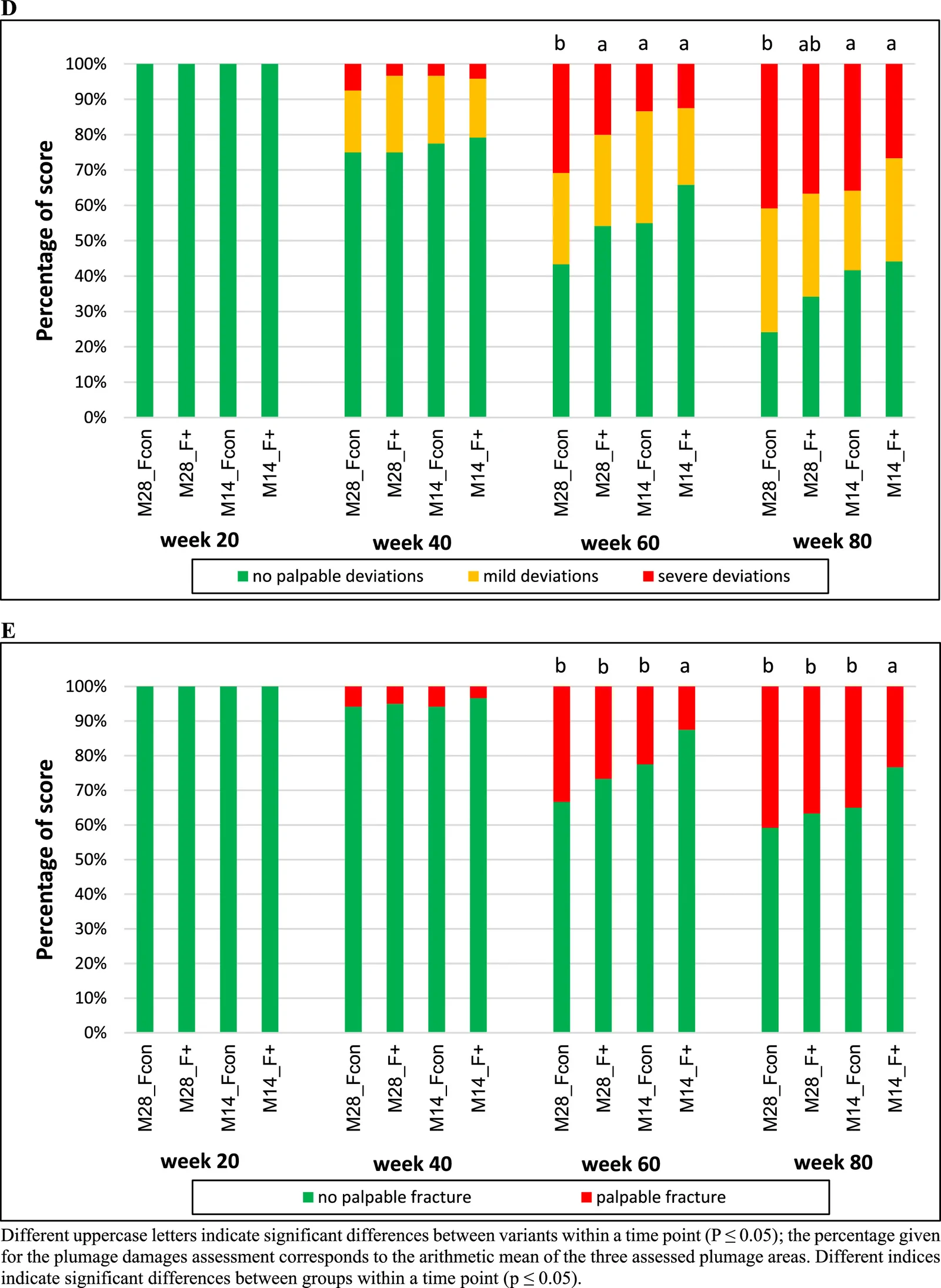
Plumage damage (A), skin injuries (B), footpad swelling (C), palpable keel bone deviations (D), and palpable keel bone fractures (E) in laying hens from four experimental groups at different ages during the laying period. Integument and keel bone characteristics are shown for hens reared with aviary opening on day 28 and day 31 receiving either standard fiber content (M28_Fcon) or moderate elevated fiber content (M28_F+), and for hens reared with aviary opening on day 14 and day 17 receiving either standard fiber content (M14_Fcon) or moderate elevated fiber content (M14_F+).

Keel bone condition was evaluated by palpation to detect keel bone deviations (KBD) and keel bone fractures (KBF), following the procedure described by Jung et al. (2020). KBD were recorded using an ordinal three-point scale (0 = intact keel bone with no or only slight lateral, dorsal, and/or ventral deviation from the straight midline ≤0.5 cm; 1 = mild deviation with lateral, dorsal, and/or ventral deviation from the straight midline >0.5 to ≤1 cm; 2 = severe deviation with lateral, dorsal, and/or ventral deviation from the straight midline >1 cm). KBF were assessed on a binary scale (0 = no palpable fracture, with no palpable callus formation or discontinuity; 1 = palpable fracture characterized by callus formation and/or discontinuity of the keel bone).

#### Statistical analyses

Microsoft Excel (Version 2021, Microsoft Corporation, Redmond, WA, USA) was used for data entry, data preprocessing, and the creation of selected graphs. All subsequent descriptive and inferential statistical analyses were performed using IBM SPSS Statistics (Version 31, SPSS Inc., Chicago, IL, USA).

For animal-based traits, individual birds were used as observational units, while compartment effects were additionally accounted for within the regression models to address potential clustering of observations within pens. Pens were considered as experimental units for performance-related parameters, whereas individual eggs served as experimental units for egg quality traits. This distinction was made to account for the hierarchical structure of the data.

Interobserver reliability for the scoring of integument traits and keel bone condition was assessed by means of a concordance analysis. Agreement between observers was quantified using the prevalence-adjusted and bias-adjusted kappa coefficient (PABAK) according to Gunnarsson et al. (2000). The interpretation of PABAK values followed the classification proposed by Landis and Koch (1977) and Kwiecien et al. (2011): ≤0.20 = poor, 0.21–0.40 = fair, 0.41–0.60 = moderate, 0.61–0.80 = good, and >0.80 = very good agreement.

The ordinally scaled integument and keel bone variables were first analyzed univariately for differences between treatment groups using the Kruskal–Wallis test (du Prel et al., 2010). When significant main effects were detected, pairwise post hoc comparisons between the four experimental variants were performed using the Mann–Whitney U test (du Prel et al., 2010).

To investigate multivariable relationships, binary logistic regression (BLR) models were applied following the approach described by Baltes-Götz (2000). Binary logistic regression was preferred over ordinal regression because some score categories contained only few observations and the proportional odds assumption required for ordinal regression was not met in all cases (McCullagh, 1980).

For this purpose, the original ordinal scoring system was converted into a binary outcome variable. Observations with a score of 0 were coded as 0 (intact condition), whereas observations with scores ≥1 were coded as 1 for SI, footpad condition, and KBF. For PD and KBD, observations with scores ≤1 were coded as 0 and observations with scores ≥2 as 1.

Within the BLR models, PD, SI, footpad condition, KBD, and KBF were used as dependent variables. Feeding during rearing, opening time of the aviary during rearing, age during the laying period, and the interaction between feeding and aviary opening time were included as explanatory variables. Compartment was included within the BLR model structure to account for potential non-independence and clustering of animals within the same pen. Multicollinearity among explanatory variables was assessed by calculating Pearson’s correlation coefficients and by performing a collinearity diagnosis using the variance inflation factor and the condition index (Menard, 1995; Field, 2013).

A Cox regression model was applied to analyze survival time in relation to animal losses, with mortality or necessary culling defined as events (Zwiener et al., 2011). Dietary treatment (Fcon, F+) and aviary opening time (M14, M28) were included as independent variables.

All statistical analyses for performance traits during laying period were performed using SAS (Version 9.4, SAS Institute Inc., Cary, NC, USA). For each response variable, descriptive statistics (mean, SD) were calculated using the *proc means* procedure. To evaluate the effects of the experimental factor, linear mixed-effects models were fitted using *proc mixed*. Fixed effect included dietary treatment and aviary opening as well as their interaction. The experimental block was included as a random effect. Model residuals were assessed for normality using qq-plots. Statistical significance was defined as p ≤ 0.05 for all inferential analyses.

## Results and discussion

### Performance, mortality, and egg quality

The performance characteristics for the four treatments are summarized in Table 1, Table 2. The performance was not influenced by the treatments (p > 0.05). The number of eggs per hen housed (380 eggs/hen), the egg mass per hen housed (23.6 kg/hen) and the shell breaking strength (53.6 N at week 44, 40.8 N in week 80 (Table 3)) were in line with the performance objectives of the breeder (Lohmann Breeders, 2021). The overall mean of the feed conversion ratio (FCR) was 2.09 kg feed per kg egg mass and was 0.06 kg feed per kg egg mass lower than the performance objectives (Lohmann Breeders, 2021). The individual egg mass was approximately 2 g lower than stated in the performance objectives. This is also reflected in the high proportion of M-sized eggs for all variants (Table 2). However, the reasons for the lower egg mass compared to the performance objectives could not be determined based on the available data. Compared to data from studies reviewed by Arulnathan et al. (2024), the FCR, the individual egg mass and the laying rates fell within the reported range. However, comparability between the present study and data of Arulnathan et al. (2024) might be compromised by the different housing systems and strains used in the reviewed studies.

**Table 1:** Effect of opening the aviary on day 14 (M14) or day 28 (M28) combined with low dietary fiber level (Fcon) or moderate elevated fiber level (F+) during pullet rearing on production traits of laying hens during the laying period (weeks 21 - 84). Given are least square means and SEM.

	**Number of eggs**		**Laying performance**		**Egg mass**		**Individual egg mass**	**ADFI**	**FCR**
	**(n)**		**(%)**		**(kg)**		**(g)**	**(g/d)**	**(kg/kg)**
	per HH	per AH	per HH	per AH	per HH	per AH			
**Variants**									
M28_Fcon	383	400	85.4	89.3	23.7	24.8	61.9	116	2.10
M28_F+	377	407	84.1	90.8	23.3	25.1	61.8	117	2.08
M14_Fcon	378	398	84.4	88.9	23.5	24.7	62.1	115	2.08
M14_F+	383	405	85.4	90.4	23.8	25.2	62.2	117	2.07
*Pooled SEM*	*8.017*	*3.706*	*1.791*	*0.826*	*0.494*	*0.215*	*0.222*	*0.768*	*0.020*
***Main Effects***									
**Feeding**									
Low fiber content	381	399	84.9	89.1	23.6	24.7	62.0	115	2.09
Moderate elevated fiber content	380	406	84.8	90.6	23.5	25.1	62.0	117	2.08
**Aviary opening day**									
Aviary opening on day 28	380	403	84.8	90.0	23.5	24.9	61.9	116	2.09
Aviary opening on day 14	380	401	84.9	89.6	23.6	24.9	62.1	116	2.08
*Pooled SEM*	*5.670*	*2.844*	*1.266*	*0.637*	*0.349*	*0.152*	*0.180*	*1.046*	*0.014*
**p-values**									
F	0.922	0.077	0.924	0.074	0.924	0.089	0.948	0.277	0.463
M	0.924	0.591	0.924	0.604	0.770	0.973	0.154	0.454	0.539
M x F	0.534	0.989	0.528	0.974	0.465	0.778	0.402	0.413	0.710

F = Fiber content, M = Management, HH = hen housed, AH = Average hen, ADFI = Average daily feed intake, FCR = Feed conversion ratio.

**Table 2:** Effect of opening the aviary on day 14 (M14) or day 28 (M28) combined with low dietary fiber level (Fcon) or moderate elevated fiber levels (F+) during pullet rearing on egg size distribution of laying hens during the laying period (weeks 21 – 84). Given are least square means and SEM.

	**Egg size distribution**				**Cracked eggs**	**Floor eggs**
	**(%)**				**(%)**	**(%)**
	**S**	**M**	**L**	**XL**		
	< 53g	53 g - < 63g	63 g - < 73g	≥ 73 g		
**Variants**						
M28_Fcon	3.5	53.5	34.9	3.8	4.3	0.07
M28_F+	4.0	54.8	34.0	3.3	3.9	0.10
M14_Fcon	3.5	52.5	35.8	4.2	4.0	0.11
M14_F+	3.1	53.2	35.5	4.0	4.2	0.05
*Pooled SEM*	*0.266*	*1.589*	*1.176*	*0.641*	*0.317*	*0.014*
***Main Effects***						
**Feeding**						
Low fiber content	4.0	35.4	53.0	3.5	4.2	0.09
Moderate elevated fiber content	3.6	34.7	54.0	3.6	4.0	0.08
**Aviary opening day**						
Aviary opening on day 28	3.5	34.5	54.1	3.8	4.1	0.09
Aviary opening on day 14	4.1	35.7	52.9	3.3	4.1	0.11
*Pooled SEM*	*0.575*	*0.832*	*1.124*	*0.189*	*0.224*	*0.010*
**p-values**						
F	0.432	0.593	0.521	0.859	0.694	0.264
M	0.205	0.340	0.440	0.107	0.948	0.727
M x F	0.761	0.797	0.853	0.131	0.359	0.019

F = Fiber content, M = Management.

**Table 3:** Effect of opening the aviary on day 14 (M14) or day 28 (M28) combined with low dietary fiber level (Fcon) or moderate elevated fiber levels (F+) during pullet rearing on egg quality parameters of laying hens. Parameters were assessed in week (wk) 44, 56, 68 and 80. Given are least square means and SEM.

	**Breaking strength**				**Albumen height**				**Albumen consistency**			
	**(N)**				**(mm)**				**(HU)**			
	wk 44	wk 56	wk 68	wk 80	wk 44	wk 56	wk 68	wk 80	wk 44	wk 56	wk 68	wk 80
**Variants**												
M28_Fcon	53.0	48.1	46.5	39.7	8.7	8.4	8.0	7.1	92.4	91.0	88.1	82.3
M28_F+	54.6	49.2	47.0	42.6	8.7	8.3	7.7	7.3	93.5	90.7	86.6	83.7
M14_Fcon	53.5	49.0	44.9	40.3	8.7	8.6	8.1	7.5	92.1	91.5	88.7	84.7
M14_F+	53.3	51.3	46.3	40.2	8.9	8.2	8.0	7.6	93.5	89.3	88.1	84.8
*Pooled SEM*	*0.998*	*0.975*	*0.917*	*0.940*	*0.092*	*0.141*	*0.119*	*0.118*	*0.479*	*0.721*	*0.659*	*0.775*
***Main Effects***												
**Feeding**												
Low fiber content	53.2	48.6	45.7	40.0	8.7	8.5 ^a^	8.1 ^a^	7.3	92.2	91.3 ^a^	88.4	83.5
Moderate elevated fiber content	53.9	50.2	46.6	41.4	8.8	8.2 ^b^	7.8 ^b^	7.4	93.1	90.0 ^b^	87.3	84.2
**Aviary opening day**												
Aviary opening on day 28	53.8	48.7	46.8	41.2	8.7	8.4	7.8 ^b^	7.2 ^b^	92.5	90.9	87.4	83.0 ^b^
Aviary opening on day 14	53.4	50.1	45.6	40.3	8.8	8.4	8.1 ^a^	7.5 ^a^	92.8	90.4	88.4	84.7 ^a^
*Pooled SEM*	*0.776*	*0.707*	*0.674*	*0.665*	*0.065*	*0.121*	*0.093*	*0.088*	*0.339*	*0.602*	*0.500*	*0.568*
**p-values**												
F	0.443	0.079	0.295	0.141	0.118	0.010	0.035	0.267	0.090	0.027	0.070	0.349
M	0.660	0.125	0.173	0.329	0.326	0.825	0.039	0.002	0.578	0.420	0.085	0.021
M x F	0.301	0.525	0.586	0.100	0.191	0.114	0.343	0.429	0.220	0.092	0.444	0.384

F = Fiber content, M = Management, HU = Haugh unit, different indices indicate significant differences within a time point (p ≤ 0.05).

The distribution of egg sizes and the proportion of cracked and floor eggs are presented in Table 2. Across all variants, size M eggs dominated, followed by size L eggs, whereas size S and XL eggs occurred only at low proportions. No differences were observed across the four treatments (all p > 0.05). Aviary management and fiber content had also no effect on the proportion of floor eggs (p > 0.05). Additional egg quality parameters, including albumen height, albumen consistency in HU and shell breaking strength, are shown in Table 3. Internal egg quality traits declined from week 44 to week 80 in all groups. This is in line with reports in literature, e.g. by Chang et al. (2024). A study involving a small number of Lohmann Brown layers from the first 33 weeks of laying showed a lower breaking strength and lower HU than the eggs in the 44th week in the present study, which underlines the overall high egg-quality, particularly in the early experimental phase (Gangnat et al., 2020). Significant differences between the feeding regimes were observed for albumen height in WA 56 and 68: hens of the Fcon group had 0.3 mm higher albumen height. This was reflected in a higher HU in WA 56 for Fcon eggs. In WA 68 and 80 the albumen height was influenced by the aviary opening. However, the changes in albumen height and HU were small compared to the changes by increasing age in Hy-Line Brown laying hens described by Chang et al. (2024). The missing physiological explanation for changes in albumen height by the variables under study and the small differences between the lsmeans suggest a random methodological bias, e.g. by selecting the analyzed eggs, rather than an actual effect of the treatments.

Descriptively, mortality was 14.5% (Fcon) and 19.3% (F+) across dietary treatments, and 16.5% (M14) and 17.4% (M28) across management groups. At the level of the four experimental variants, mortality rates were 15.0% (M28_Fcon), 19.7% (M28_F+), 14.0% (M14_Fcon), and 19.0% (M14_F+). Cox regression revealed no effect of aviary opening time (P = 0.591), but a significant effect of diet (P = 0.015), with higher survival probability in Fcon than in F+.

Cannibalism-related mortality was 7.0% in Fcon and 8.9% in F+, and 7.9% in M14 and 8.0% in M28. At the variant level, values were 6.8% (M28_Fcon), 9.2% (M28_F+), 7.1% (M14_Fcon), and 8.7% (M14_F+). Neither Kaplan–Meier analysis (P ≥ 0.127) nor Cox regression (P ≥ 0.129) showed effects of diet, management, or their combination.

According to the German KTBL guideline, cumulative mortality should not exceed 16.0% by 84 weeks (Knierim et al., 2020). This threshold was exceeded in several groups, indicating elevated mortality. About half of all losses were cannibalism-related. Cannibalism is a major cause of mortality in non–beak-trimmed flocks, although mortality is influenced by multiple interacting factors, including diseases, housing, nutrition, genetics, and management (Aerni et al., 2005; Fossum et al., 2009). Future studies should determine the cause of death for each hen to better assess mortality patterns and their relation to rearing interventions.

The dietary effect on survival indicates that increased fiber during rearing did not yield consistent long-term benefits. Although fiber-rich diets promote foraging and longer feeding times (van Krimpen et al., 2005; Svihus, 2011), this did not improve survival during lay. Fiber strategies during rearing should therefore be considered together with feeding during lay (Mens et al., 2020). The common laying diets contained 5.0% crude fiber, compared with 7.5–8.1% in the F+ and 5.7–5.8% in the Fcon developer diets. Thus, the transition to the laying diet involved a marked reduction in dietary fiber concentration particularly for hens previously fed F+. Whether this dietary transition contributed to the observed differences in survival cannot be determined from the present study.

Together with the contrasting effects on plumage damage and KBD described below, this indicates that carry-over effects of dietary interventions during rearing may be trait-specific rather than uniformly beneficial.

### Integument and keel bone condition

The agreement between the two observers involved in the scoring of the key integument traits and keel bone condition was assessed using concordance analysis. High PABAK values ranging from 0.83 to 0.97 were obtained for all traits assessed. According to the classification of Landis and Koch (1977), this corresponds to almost perfect agreement between observers and indicates a high reliability of the applied scoring schemes.

The BLR models, which were used as the main analytical approach in this study, evaluated the effects of fiber content in the rearing diet, the timing of aviary opening during rearing, age during the laying period, and the interaction between diet and aviary opening on PD, SI, footpad condition, KBD, and KBF during the laying period (Table 4). Effects of rearing diet were observed for PD and KBD (P ≤ 0.042), whereas effects of aviary opening timing were found for PD, SI, and KBD (P ≤ 0.004). Age significantly affected all traits (P < 0.001). No significant interaction between dietary fiber content and aviary opening timing was detected for any trait (P ≥ 0.115).

**Table 4:** Effects of dietary fiber level and aviary opening day during pullet rearing, age during the laying period, and their interaction on plumage damage, skin injuries, footpad swelling, keel bone deviations, and keel bone fractures in brown layer hybrids during the laying period – results of binary logistic regression models.

**Trait**	**Score 1 (%)**	**Coefficients (SE)**	**Odds ratio (95% CI)**	**p-value**
**Plumage damage**				
				
*Feeding*				
Low fiber content	64.6	Reference	Baseline	
Moderate elevated fiber content	67.8	0.71 (0.24)	2.04 (1.27-3.28)	**0.003**
*Aviary opening day*				
Aviary opening on day 28	59.7	Reference	Baseline	
Aviary opening on day 14	53.3	−1.09 (0.26)	0.33 (0.20-0.56)	**<0.001**
*Age*		0.18 (0.01)	1.20 (1.18-1.22)	**<0.001**
*Feeding x Aviary opening day*		0.32 (0.35)	1.38 (0.69-2.75)	0.356
Intercept		−8.08 (0.42)		
**Skin injuries**				
*Feeding*				
Low fiber content	18.9	Reference	Baseline	
Moderate elevated fiber content	16.1	−0.19 (0.16)	0.82 (0.58-1.14)	0.239
*Aviary opening day*				
Aviary opening on day 28	20.8	Reference	Baseline	
Aviary opening on day 14	14.2	−0.50 (0.17)	0.60 (0.43-0.85)	**0.004**
*Age*		0.04 (0.00)	1.04 (1.03-1.05)	**<0.001**
*Feeding x Aviary opening day*		−0.03 (0.25)	0.97 (0.58-1.61)	0.921
Intercept		−3.46 (0.23)		
**Footpad swelling**				
*Feeding*				
Low fiber content	18.3	Reference	Baseline	
Moderate elevated fiber content	20.3	0.32 (0.16)	1.37 (0.99-1.90)	0.057
*Aviary opening day*				
Aviary opening on day 28	19.9	Reference	Baseline	
Aviary opening on day 14	18.7	0.12 (0.12)	1.12 (0.80-1.57)	0.495
*Age*		0.03 (0.00)	1.03 (1.02-1.03)	**<0.001**
*Feeding x Aviary opening day*		−0.37 (0.23)	0.68 (0.43-1.09)	0.115
Intercept		−2.94 (0.20)		
**Keel bone deviations**				
*Feeding*				
Low fiber content	16.8	Reference	Baseline	
Moderate elevated fiber content	13.4	−0.37 (0.18)	0.69 (0.48-0.99)	**0.042**
*Aviary opening day*				
Aviary opening on day 28	17.9	Reference	Baseline	
Aviary opening on day 14	12.3	−0.59 (0.19)	0.55 (0.38-0.80)	**0.002**
*Age*		0.06 (0.01)	1.06 (1.05-1.07)	**<0.001**
*Feeding x Aviary opening day*		0.16 (0.28)	1.17 (0.67-2.03)	0.578
Intercept		−4.75 (0.29)		
**Keel bone fractures**				
*Feeding*				
Low fiber content	17.9	Reference	Baseline	
Moderate elevated fiber content	13.4	−0.23 (0.18)	0.79 (0.55-1.13)	0.200
*Aviary opening day*				
Aviary opening on day 28	18.5	Reference	Baseline	
Aviary opening on day 14	12.8	−0.34 (0.18)	0.71 (0.49-1.02)	0.065
*Age*		0.06 (0.00)	1.06 (1.05-1.07)	**<0.001**
*Feeding x Aviary opening day*		−0.39 (0.28)	0.67 (0.38-1.16)	0.157
Intercept		−4.81 (0.29)		

SE – standard error, CI – confidence interval; Score 0 — intact condition of the integument or keel bone, score 1 — changes in the condition of the integument or keel bone.

The distribution of the individual scoring categories for PD, SI, footpad condition, KBD, and KBF is presented in Figure 1.

Plumage condition deteriorated markedly with increasing age of the hens. Across all four variants, the proportion of hens with intact plumage decreased from 99.4% at 20 WA to 67.8% at 40 WA, 20.8% at 60 WA, and finally 16.5% at 80 WA.

The BLR analysis showed that both dietary fiber content during pullet rearing (P = 0.003) and aviary opening day during rearing (P < 0.001) significantly affected the risk of PD. Hens from variants with increased dietary fiber content showed a higher risk of PD than hens from the control feeding treatment. At the same time, the risk of PD was lower in M14 hens than in M28 hens.

In the univariate analysis comparing the four variants within each age group, significant differences in the prevalence of PD were observed at 40, 60, and 80 WA (each P < 0.001), but not at 20 WA (P = 0.255). At 40 WA, hens of the M14_Fcon variant showed the lowest prevalence of PD, whereas the highest prevalence of plumage damage was observed in the M28_F+ variant; the two remaining variants showed intermediate values. A similar pattern was observed at 60 WA, where M14_Fcon again showed a lower prevalence of PD than the other variants. At 80 WA, plumage damage was more pronounced—independent of the feeding treatment—when the rearing aviary had been opened on day 28 rather than day 14.

The more favorable long-term plumage condition following earlier aviary opening supports the assumption that early access to a structured litter environment promotes behavioral development in a way that reduces the risk of feather pecking during the laying period (Janczak and Riber, 2015; de Haas et al., 2014). This is consistent with studies demonstrating long-term effects of rearing conditions on plumage damage (Casey-Trott et al., 2017; Schreiter et al., 2020a). This is further supported by studies demonstrating that early access to litter and environmental enrichment during rearing can reduce feather pecking and feather damage later in life (Tahamtani et al., 2016). Importantly, the present findings extend previous evidence by demonstrating that not only fundamentally different rearing environments, but also the timing of a specific management intervention within the same aviary system can have persistent effects on integument condition into late lay. However, with regard to practical implementation, it remains to be clarified to what extent very early aviary opening can be applied under commercial conditions in large flocks.

In contrast, the higher PD prevalence observed with increased dietary fiber during rearing was unexpected. As fiber-rich diets primarily act via behavioral mechanisms (Mens et al., 2020), it is likely that these effects were not consistently transferred into the laying period. In addition, differences in feed intake and body weight at the end of rearing (Schreiter et al., 2026) may have affected the birds’ ability to cope with the transition to lay (de Haas et al., 2014). Furthermore, the marked reduction in dietary fiber concentration after transfer, particularly in hens previously fed F+, may be relevant when interpreting the subsequent differences in plumage condition. However, a direct effect of this dietary transition cannot be established from the present study. Thus, the present findings indicate that nutritional interventions during rearing may result in trait-specific carry-over effects rather than uniformly beneficial long-term responses.

The proportion of hens with SI increased markedly during the laying period. Across all assessed hens, 0.8% (20 WA), 9.8% (40 WA), 28.5% (60 WA), and 29.4% (80 WA) showed mild or severe SI.

The BLR models identified aviary opening day during rearing as a significant factor affecting the risk of SI (P = 0.004). M14 hens showed a lower risk of SI than M28 hens, whereas dietary fiber content during rearing had no detectable effect (P = 0.239). In the univariate analysis, differences between variants were observed only at 80 WA (P < 0.001). At this age, SI occurred less frequently in hens from groups with earlier aviary opening (M14) than in hens from groups with later aviary opening (M28). The fact that aviary opening time also affected SI highlights the functional linkage in the underlying causes of plumage damage and skin injuries in flocks affected by feather pecking and cannibalism (Nicol et al., 2013; Rodenburg et al., 2013). Accordingly, a more favorable plumage condition resulting from earlier access to a litter area may indirectly reduce the risk of secondary skin injuries.

Moderate changes in footpad condition were observed over the course of the laying period. Across all assessed hens, 0.0% (20 WA), 27.1% (40 WA), 19.6% (60 WA), and 30.0% (80 WA) of the hens showed mild or severe footpad swelling. No differences in the prevalence of footpad swelling between the four variants were detected in the univariate analysis (P ≥ 0.257). Similarly, the BLR models did not detect a significant effect of dietary fiber content during pullet rearing (P = 0.057) or aviary opening day during rearing (P = 0.495) on the risk of footpad swelling.

As expected, keel bone condition also deteriorated with advancing age during the laying period. The proportion of hens with palpable KBD (mild or severe) increased across all assessed hens from 0.0% (20 WA) to 23.3% (40 WA), 45.4% (60 WA), and 64.0% (80 WA). At the same time, the proportion of hens with palpable KBF increased with age and amounted to 0.0% (20 WA), 5.0% (40 WA), 23.8% (60 WA), and 34.0% (80 WA).

The BLR models showed that the occurrence of KBD was significantly associated with both dietary fiber content during pullet rearing (P = 0.042) and aviary opening day during rearing (P = 0.002). Hens from variants with increased dietary fiber content showed a lower risk of KBD than hens from variants with standard fiber content. Likewise, the risk of KBD was lower in M14 hens than in M28 hens, whose rearing aviary had been opened later. In contrast, no effects of dietary treatment (P = 0.200) or aviary opening day during rearing (P = 0.065) were detected for KBF.

In the univariate analysis, differences in the prevalence of KBD between variants were observed at 60 WA (P = 0.001) and 80 WA (P = 0.015). At 80 WA, the M28_Fcon variant showed a significantly higher prevalence of KBD than both M14 variants. A similar pattern was already observed at 60 WA, where the M28_Fcon variant appeared indifferent compared with the remaining variants.

KBF occurred less frequently in hens of the M14_F+ variant at 60 WA (P = 0.002) and 80 WA (P = 0.029) than in the other three variants. Accordingly, hens of this variant showed numerically the lowest prevalence of both KBD (29.2% mild and 26.7% severe deviations) and KBF (23.3%) at 80 WA..

Methodologically, it should be noted that keel bone damage in the present study was assessed by palpation only, which has lower diagnostic accuracy than imaging techniques (Casey-Trott et al., 2015; Buijs et al., 2019). The generally consistent, although not always significant, effects of rearing diet and management on KBD and KBF suggest that both traits are influenced by similar factors, while differing in their expression. This aligns with the assumption that keel bone deviations and fractures are related but do not share identical underlying mechanisms (Rufener and Makagon, 2020; Li et al., 2024).

The lower prevalence of KBD in hens with earlier aviary opening indicates that early access to complex structures and increased movement opportunities may promote motor development and improve the use of the aviary system. Studies have shown that increased activity and structural complexity during rearing are associated with improved bone development and adaptation to multi-tier systems (Casey-Trott et al., 2017) and may also influence keel bone damage (Rentsch et al., 2024). This may reduce uneven loading and limit the development of deviations. The persistence of these effects until 80–84 WA extends previous longitudinal evidence on rearing-related effects into a production-relevant extended laying cycle.

It should be noted that the present study evaluated hens until 84 weeks of age. Although this represents an extended laying cycle compared with conventional production systems, current production concepts increasingly target laying periods beyond 90 weeks (Damme et al., 2023). Future studies should therefore examine whether the observed carry-over effects persist throughout these longer production cycles (European Commission, 2021; Kjaer and Bessei, 2013; Welfare Quality®, 2009).

## Conclusion

The present study demonstrates that specific, practically manageable interventions during pullet rearing can exert persistent and trait-specific carry-over effects into an extended laying cycle. Earlier aviary opening was associated with more favorable long-term integument and keel bone condition without impairing subsequent performance. Thus, even the timing of aviary opening within an otherwise comparable rearing system can have long-term consequences. However, the feasibility of aviary opening from day 14 in large commercial flocks requires further evaluation.

In contrast, moderately increased dietary fiber during rearing did not provide a consistent long-term benefit: a lower risk of KBD was accompanied by greater plumage damage and lower survival probability. These findings demonstrate that carry-over effects of rearing interventions can be intervention- and trait-specific and partly contrasting. Their persistence until 80–84 WA under identical conditions during lay emphasizes the importance of targeted and phase-integrated evaluation of rearing strategies for extended laying cycles.

## Authorship contribution statement

R. Schreiter conceived and designed the study, coordinated the experimental work, participated in data collection, performed the statistical analyses of integument and keel bone traits, interpreted the results, prepared the corresponding figures and tables, and drafted and revised the manuscript.

S. Becker analyzed the performance data, prepared the corresponding figures and tables, interpreted the results, and wrote and revised the performance-related sections of the manuscript.

L. Fitz performed integument and keel bone assessments, processed the corresponding experimental data, and critically reviewed the manuscript for important intellectual content.

J. Krieg contributed to the planning of the performance data analysis, analyzed and interpreted the performance data, and wrote and revised the corresponding sections of the manuscript.

P. Schulte-Huxel contributed to the planning of the performance data analysis, analyzed and verified the performance results, and wrote and revised the corresponding sections of the manuscript.

A. Schröder contributed to the experimental design and data collection during the rearing period, processed the corresponding experimental data, and critically reviewed the manuscript for important intellectual content.

J. Slama contributed to the design and implementation of the feeding experiment, performed feed analyses, processed the corresponding experimental data, and critically reviewed the manuscript for important intellectual content.

H. Louton contributed to the experimental design, particularly the selection and definition of keel bone health indicators, provided scientific guidance on data collection and assessment procedures, contributed to the interpretation of the findings, and critically reviewed the manuscript for important intellectual content.

P. Hofmann contributed to the experimental design, particularly the selection and definition of performance parameters, secured funding and provided the resources required for the study, contributed to the interpretation of the results, and critically revised the manuscript for important intellectual content.

All authors critically reviewed the manuscript for important intellectual content, reviewed and explicitly approved the final version, and agree to be personally accountable for all aspects of the work, ensuring that any questions regarding its accuracy or integrity are appropriately investigated and resolved.

## Declaration of Ai and Ai-assisted technologies in the writing process

In the preparation of this manuscript, the authors used DeepL and ChatGPT (OpenAI) to support translation, grammar correction, and linguistic refinement. All content was subsequently reviewed and revised by the authors, who take full responsibility for the final version of the manuscript.

## Disclosures

The authors have no conflicts of interest to declare.
